# TECHNICAL SUPPORTS TO BRIDGE THE DIGITAL DIVIDE FOR OLDER ADULTS PARTICIPATING IN TELEMEDICINE

**DOI:** 10.1093/geroni/igac059.022

**Published:** 2022-12-20

**Authors:** Lauren Moo, Andrew Nguyen, Kendra Pugh, Jaye McLaren, Steven Shirk, Maureen O'Connor

**Affiliations:** VA Bedford Health Care System, Bedford, Massachusetts, United States; Bedford VA Research Inc, Boston, Massachusetts, United States; VA Bedford Healthcare System, Bedford, Massachusetts, United States; VA Bedford Healthcare System, Bedford, Massachusetts, United States; VA Bedford Healthcare System, Bedford, Massachusetts, United States; VA Bedford Healthcare System, Bedford, Massachusetts, United States

## Abstract

Older adults’ relative lack of technological literacy is a barrier to telemedicine, including participation in virtual caregiver support programs. Details of technology assistance required before and during a virtual, seven-session group skills training program for family caregivers were recorded. A majority of participants, all older adults, had difficulty using videoconferencing technology that ranged from finding meeting links in emails to difficulty with basic device use. Personalized support was provided to each caregiver as needed. None required any support by the final session. Those with initial technology challenges felt a sense of accomplishment and increased tech comfort by the end of the sessions. While some older adults had difficulty using telemedicine technology, they were eager to learn and educable with personalized help. Infrastructure to provide this personalized help is necessary to support older adults’ desire to engage in telemedicine and to reduce the digital divide.

